# Expression of Immune Response Markers in Arab Patients With Lung Cancer

**DOI:** 10.1200/GO.20.00107

**Published:** 2020-08-04

**Authors:** Abdul Rahman Jazieh, Adda Bounedjar, Hanaa Bamefleh, Turki Alfayea, Hatim Q. Almaghraby, Ayed Belarabi, Wahiba Ouahioune, Zoubir Derbouz, Mohammad Alkaiyat, Khaled Alkattan, Moussab Damlaj, Walid E. Khalbuss

**Affiliations:** ^1^ Department of Oncology, King Abdulaziz Medical City, King Abdullah International Medical Research Center, King Saud bin Abdulaziz University for Health Sciences, Ministry of National Guard Health Affairs, Riyadh, Kingdom of Saudi Arabia; ^2^ Universite Blida1 Laboratoire de Cancerologie, Faculte De Medicine, Blida, Algeria; ^3^ Oncology Department, Princess Nourah Cancer Center, King Saud bin Abdulaziz University for Health Sciences, Ministry of National Guard Health Affairs, Jeddah, Kingdom of Saudi Arabia; ^4^ Department of Pathology, King Abdulaziz Medical City, King Saud bin Abdulaziz University for Health Sciences, Ministry of National Guard Health Affairs, Jeddah, Kingdom of Saudi Arabia; ^5^ College of Medicine, Alfaisal University, Riyadh, Kingdom of Saudi Arabia

## Abstract

**PURPOSE:**

Programmed death-ligand 1 (PD-L1) is a marker for checkpoint inhibitor use in the management of solid tumors, especially in non–small-cell lung cancer (NSCLC). Our study was aimed at determining the patterns of PD-L1 expression and cluster of differentiation 8 (CD8) immunostains in patients with NSCLC in the Arab population.

**METHODS:**

Archival tumor tissue from patients with a confirmed diagnosis of NSCLC were obtained and stained for PD-L1 with antibody 22C3, using immunohistochemistry staining and giving the tumor proportion score (TPS) as a percentage from 0%-100% of stained tumor cells. Tumors were categorized into negative expressers (TPS < 1%), low positive (TPS, 1%-49%), and high positive (TPS, 50%-100%). Correlation of expression with clinical and pathologic features, including CD8-positive (CD8+) lymphocyte density, was also analyzed.

**RESULTS:**

Two hundred patients with NSCLC were included in the study from 6 centers in Saudi Arabia and Algeria. Median age was 65 years (28-93 years), and the majority were men (75%) with stage 4 NSCLC (64%). The TPS was high in 37 patients (18%), low in 60 patients (30%), and negative in 103 patients (52%). In a univariate analysis, the following were significant predictors of any PD-L1 expression (> 1%): male sex, being Saudi national patients, high expression of CD8+, and presence of tumor-infiltrating lymphocytes. In the multivariate analysis, only high expression of CD8+ cells (≥ 2+) was significant, with an odds ratio of 4.4 (95% CI, 1.5 to 12.9; *P =* .003)

**CONCLUSION:**

PD-L1 expression in our population is similar to the published literature and correlated with the density of CD8+ cells. Validation of the predictive value of this marker in our population and identifying easier and reliable methods to test for it are warranted.

## INTRODUCTION

Checkpoint inhibitors have emerged as effective cancer therapies that have a unique mechanism of action, tolerable toxicity profile, and efficacy across many tumor types.^1-4^ They are approved for the treatment of many solid tumors, including non–small-cell lung cancer (NSCLC), melanoma, renal cell cancer, and others.^5^

**CONTEXT**

**Key Objective**
To our knowledge, this study is the first in our patient population to describe the pattern of programmed death-ligand 1 expression and the correlation with cluster of differentiation 8–positive cells in patients with lung cancer in the Middle East and North Africa region.
**Knowledge Generated**
This study will add to the literature new knowledge about the subject in different patient populations that were not studied before.
**Relevance**
This study will help increase the awareness of oncologists in the region about the use of markers and, most importantly, use of checkpoint inhibitors in their patients and makes the topic relevant to their practice settings.

NSCLC is the leading cause of cancer-related deaths globally, with 1.59 million deaths annually.^6^ Historically, lung cancer is a fatal disease in its advanced stages, because systemic therapy does not have a major impact on the long-term survival of these patients. The addition of checkpoint inhibitors to the armamentarium to fight this deadly disease has had a great effect on disease management and changed the standard of care. Although the toxicity profile is favorable compared with chemotherapy, patient selection is critical to identify the individual patients who will benefit the most from these agents and, therefore, to avoid ineffective treatment that may be associated with physical and financial toxicities.

Programmed death-ligand 1 (PD-L1) is a useful predictive marker of response for different checkpoint inhibitors and diseases.^7^ Specifically, the expression of PD-L1 in NSCLC was identified as a useful biomarker to predict benefits from checkpoint inhibitors, namely, pembrolizumab, and the US Food and Drug Administration (FDA) approval of this medication for lung can-cer was based on the expression of PD-L1. In October 2015, the FDA approved pembrolizumab for second-line treatment of NSCLC with a companion diagnostic, the PD-L1 immunohistochemistry 22C3 pharmDx test (Agilent Technologies, Santa Clara, CA), which was the first test designed to detect PD-L1 expression in NSCLC tumors.^8^ Treatment of patients with PD-L1–expressive metastatic NSCLC resulted in an impressive 5-year survival of 30% in first-line and 25% in second-line treatment, representing a major improvement in patient outcomes.^9^ Furthermore, PD-L1 expression was a predictor of better clinical outcome for the treatment of NSCLC compared with chemotherapy alone, even in patients with a low PD-L1 tumor proportion score (TPS).^10^

PD-L1 expression has been reported in various tumor types and different populations.^11-13^ Currently, there are no data available about the expression of PD-L1 in solid tumors in the Arab population, especially in lung cancer. The purpose of this study was to determine the prevalence of PD-L1 tumor cell expression and cluster of differentiation 8–positive (CD8+) tumor-infiltrating lymphocytes (TILs) in NSCLC in the Arab population and correlate it with various demographic, clinical, and pathologic features.

## METHODS

This was a retrospective study using patient medical records and archival tissues of patients with lung cancer from participating centers. Inclusion criteria were any adult patients > 18 years of age, with histologic confirmation of NSCLC, with any TNM stage, and with available and sufficient tissue sample for PD-L1 testing (> 100 viable tumor cells). Consecutive patients were selected to avoid selection bias. We excluded any patients with samples that were subject to the decalcification process or insufficient tissue to perform the test.

Sites had submitted 10 unstained slides per patient for central testing at central laboratory, with clinical research forms capturing demographic, disease, and clinical data. Approval of the institutional review boards was obtained before starting the study.

### PD-L1 Staining

Tissue samples were stained for PD-L1 with the 22C3 pharmDx Kit on the Dako Autostainer Link 48 platform (Agilent Technologies, Santa Clara, CA). Deparaffinization, rehydration, and target retrieval procedures were performed using EnVision FLEX Target Retrieval solution (1×, low pH) and EnVision FLEX wash buffer (1×; Agilent Technologies). The tissue samples were then placed on the Autostainer Link 48. This instrument performed the staining process by applying the appropriate reagent, monitoring incubation time, and rinsing slides between reagents. The reagent times were preprogrammed in the Dako Link software. A sample with the primary antibody omitted was used as a negative control. Samples were subsequently counterstained with hematoxylin and mounted in nonaqueous, permanent mounting media. The stained slides were evaluated by pathologists, and the TPS was given for each patient. TPS was defined as the percentage of viable tumor cells with any perceptible membrane staining irrespective of staining intensity. Normal cells and tumor-associated immune cells were excluded from scoring. Each patient was divided into 1 of 3 levels based on TPS: < 1% (no PD-L1 expression), 1%-49% (low PD-L1 expression), or ≥ 50% (high PD-L1 expression).

### Immunohistochemical Staining of CD8 and Evaluation (scoring)

Sections 4 μm in thickness for immunohistochemistry were cut from the 73 patients with available tissue, deparaffinized, and dehydrated. For antigen retrieval, sections were treated with 0.01 M citrate buffer (pH, 6.0) for 5 minutes in a microwave oven, followed by treatment with 3% H_2_O_2_ to quench endogenous peroxidase. Sections were then treated with the normal serum of the secondary antibody to block nonspecific binding and then incubated with anti-CD8 (Clone C8/144B; Dilution 1:50; Cell Marque, Rocklin, CA). Immunohistochemical staining was conducted following a compact polymer method using a Ventana medical system Benchmark ULTRA and Ultra View DAB detection system (Ventana Medical Systems, Tucson, AZ). Only CD8+ lymphocytes intimately admixed with tumor cells were evaluated and were scored as negative if none were seen, +1 if few (1-5) were seen in high-power field, +2 if a moderate number (6-15) were seen in high-power field, and +3 if a marked number (> 15) were seen in high-power field.

### Statistical Analysis

Baseline patient information, disease, and clinically related variables were reported for the entire cohort and stratified per PD-L1 (positive *v* negative) using descriptive statistics (counts, medians, and percentages). Categorical and continuous variables were compared using Pearson's χ^2^ and Wilcoxon tests as appropriate. Univariate analysis was computed using nominal logistic regression to predict factors associated with PD-L1 expression and patient characteristics, such as age, sex, and smoking history, as well as various tumor factors, such as stage, histologic subtype, and TIL. Multivariate logistic regression was subsequently computed by incorporating any variable with a *P* ≤ .05. Statistical analysis was performed using JMP Pro Version 11 (SAS Institute, Cary, NC) software.

## RESULTS

Two hundred consecutive patients with NSCLC were enrolled from 6 medical facilities in Saudi Arabia and Algeria. Median age was 65 years (28-93 years), 75% of patients were men, and the majority had adenocarcinoma and were stage IV (Table 1).

**TABLE 1:**
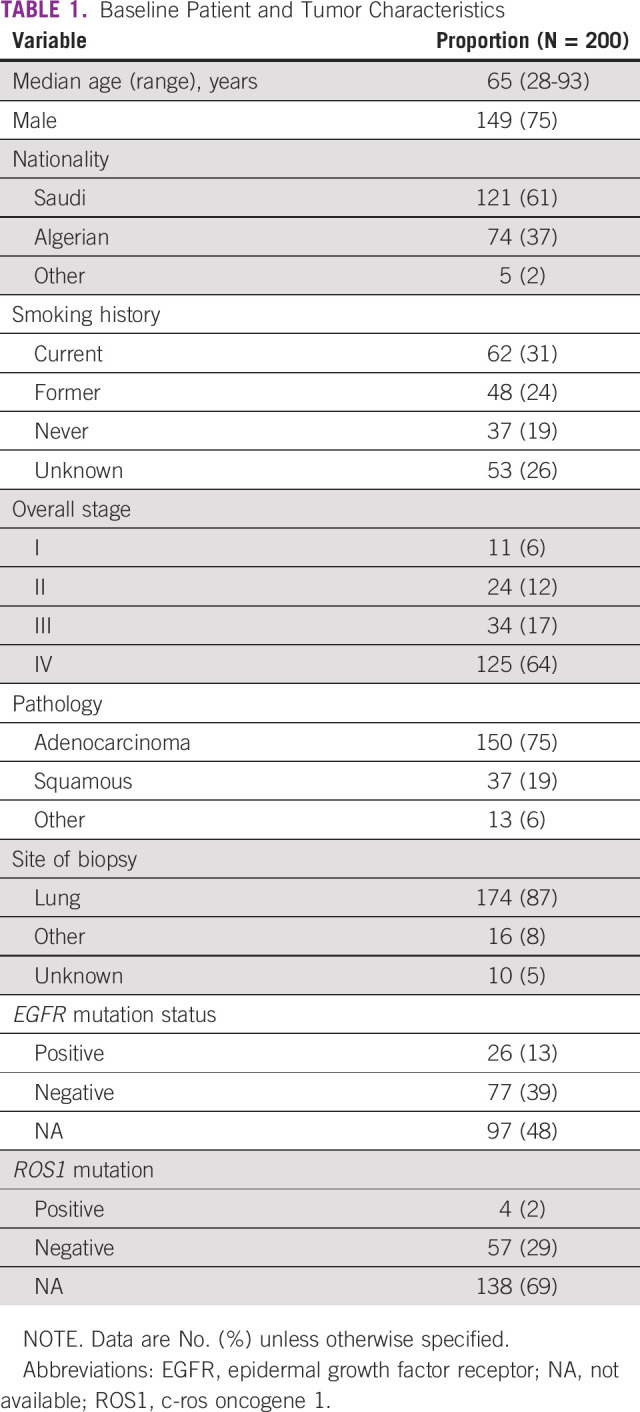
Baseline Patient and Tumor Characteristics

The TPS was high in 37 patients (18%), low in 60 patients (30%), and negative in 103 patients (52%; Table 2). CD8+ cells were tested in the 73 patients with available tissue and were found to be negative in 12 specimens (18%), +1 in 39 specimens (53%), and strongly positive +2 and +3 in 21 specimens (29%; Table 2).

**TABLE 2:**
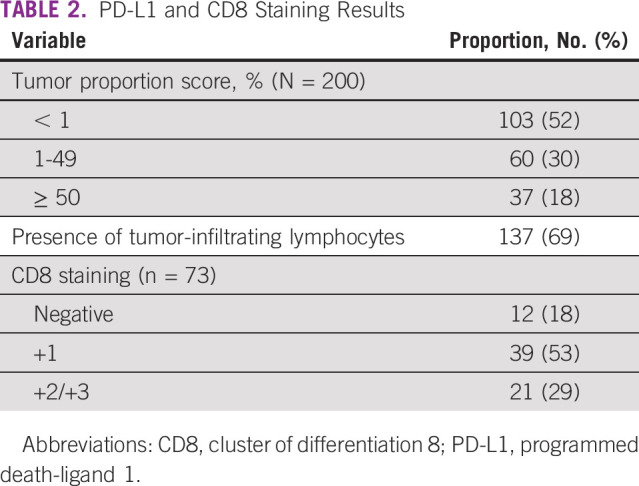
PD-L1 and CD8 Staining Results

**FIG 1:**
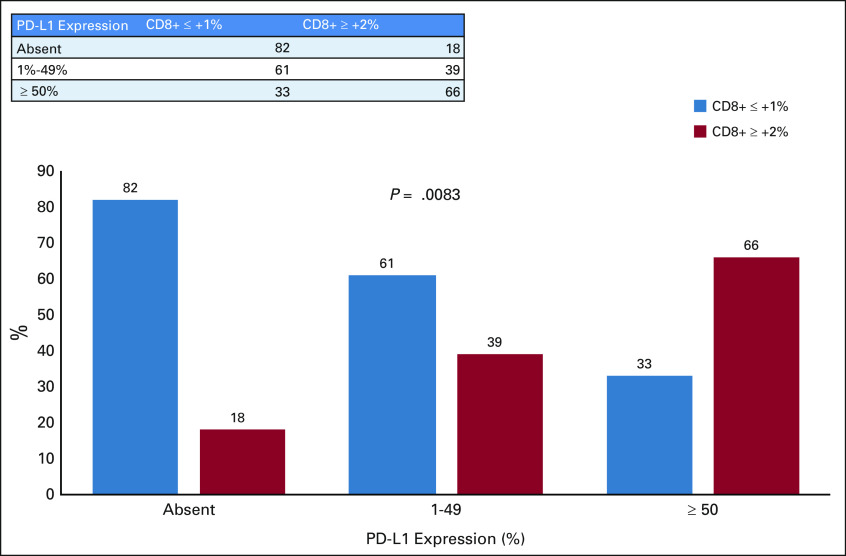
Programmed death-ligand 1 (PD-L1) expression correlated with the density of cluster of differentiation 8–positive (CD8+) cells.

In the univariate analysis, the following were significant predictors of any PD-L1 expression (> 1%): female sex, being Saudi patients, high-grade tumor, high expression of CD8, and the presence of TILs (Tables 3 and 4). However, in the multivariate analysis, a high expression of CD8 (≥ 2+) was highly significant, with an odds ratio (OR) of 11.2 (95% CI, 1.94 to 64.6; *P* = .003), and there was a trend of significance for the presence of TILs, with an OR of 5.9 (95% CI 0.58 to 61; *P =*. 08). The PD-L1 expression correlated with the density of CD8+ cells (Fig 1).

**TABLE 3:**
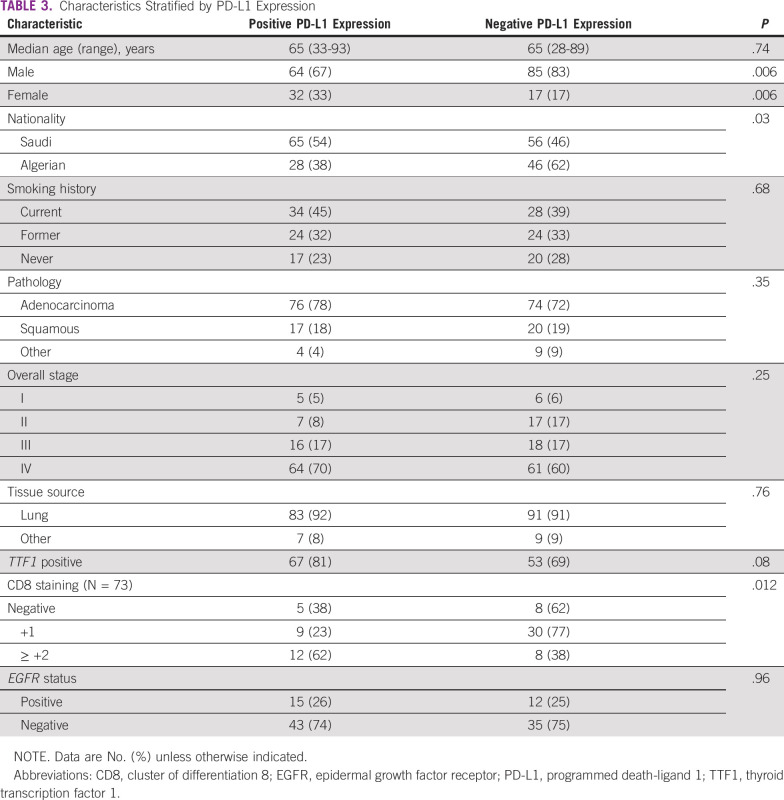
Characteristics Stratified by PD-L1 Expression

**TABLE 4:**
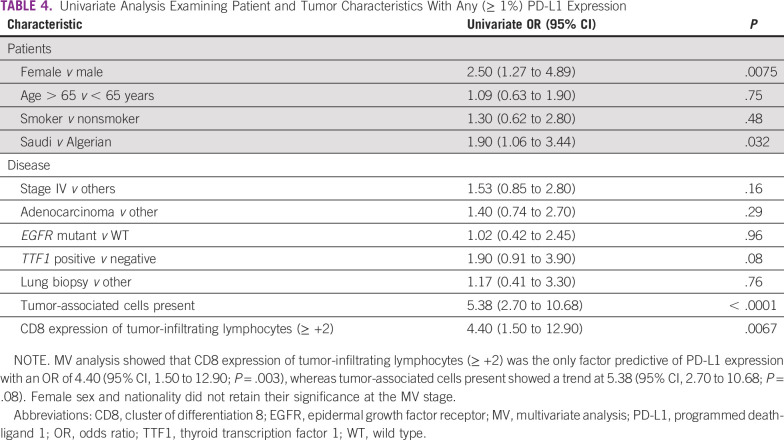
Univariate Analysis Examining Patient and Tumor Characteristics With Any (≥ 1%) PD-L1 Expression

## DISCUSSION

Our study revealed the level of PD-L1 expression in our patient population to be negative in approximately half of the patients and strongly positive in only 18%. The prevalence was similar to figures that have been reported by different investigators. In a large study of 2,617 patients recruited from 18 countries, PD-L1 > 50% was found in 22%, 52% of patients had PD-L1 > 1%, and 48% had PD-L1 < 1%; with similar prevalence in different populations from different geographical areas,^11^ this is also reflected in our findings within our patient population.

In 1,071 Asian patients with surgically resected NSCLC, 33.7% had a prevalence of PD-L1 > 1%, and only 10.8% had PD-L1 > 50%. Expression of PD-L1 was higher in squamous cell carcinoma than in adenocarcinoma. In the adenocarcinoma subgroup, PD-L1 expression on tumors was higher in males and smokers, and in patients with high histologic grade, with relatively high TNM status, with advanced American Joint Commission on Cancer stage, and positive for ALK rearrangement. However, epidermal growth factor receptor–mutated tumors showed relatively lower PD-L1 expression compared with wild-type patients.^12^

Our study revealed a correlation with certain demographic variables, such as female sex and being Saudi versus Algerian. Other results are different in terms of prevalence based on sex, because some studies have reported greater prevalence of PD-L1 expression in males, whereas others have not found a sex difference.^12,14^ We do not have a scientific explanation regarding the differences between Saudi and Algerian patients, but they may be related to the difference in tumor biology and causes between the 2 populations. The only difference between the 2 populations was that there was a higher fraction of patients with advanced stage in the Saudi population. Sex and nationality were not significant factors in the multivariate analysis.

The correlation between PD-L1 expression and patient and disease characteristics was not consistent across studies because of the different patient populations studied and the different reagents used; most important were the limitations related to PD-L1 testing in general.

In our study, there was a significant positive correlation with CD8+ cells and PD-L1 expression. Other authors reported increased expression of PD-L1 with densities of CD8+ cells in gastric and gastroesophageal junction tumors, hepatocellular carcinoma,^15^ and synovial sarcoma.^16^ In NSCLC, the expression of PD-L1 and CD8+ cells was studied by different investigators to evaluate the correlation between them and their predictive values. For example, multiple studies in patients with NSCLC revealed that patients with tumors positive for CD8+ and with PD-L1 negative expression had better survival.^17-20^

One study stratified 136 patients with resected NSCLC into 2 prognostic groups: group 1 (CD8+/PD-L1-negative) versus group 2 (CD8/PD-L1: positive/positive, negative/negative, and negative/positive). Group 1 had better overall survival (median, not reached [NR] *v* 29.4 months) and relapse-free survival (median, NR *v* 17.6 months) compared with group 2.^17^

Despite the availability of other immune markers, PD-L1 remains the most important marker for clinical practice to date because it guides the management of first-line patients with NSCLC. However, PD-L1 testing in tissue has many limitations, mainly related to tumor heterogeneity, in addition to the difficulty in accessing the tissue and the differences between the tumor and metastatic sites.^18,21,22^

Therefore, testing for PD-L1 in the blood (liquid biopsy) and in circulating tumor cells may give a better idea about the tumor and will be easily accessible for repeated testing and patient monitoring.^23,24^ Furthermore, it was reported that PD-L1 detection in peripheral blood was associated with worse survival of NSCLC,^25^ even in patients treated with checkpoint inhibitors.^26^ The techniques and use of liquid biopsy to evaluate biologic markers for immunotherapy including checkpoint inhibitors has great potential because of its convenience, safety, cost effectiveness, and ability to be performed repeatedly.^27^

Our study has the limitations inherent in a retrospective design, such as missing data, for example, smoking. We did not have an adequate number of patients treated with checkpoint inhibitors to correlate PD-L1 expression with response and outcome. The study did not calculate the prognostic value of these markers, which would have added value to our outcomes if performed, although it was not part of the study objectives. These issues should be tackled with future longitudinal prospective studies with adequate follow-up.

In conclusion, our study revealed a positive PD-L1 prevalence in more than half of our patient population, with 18% expressing TPS ≥ 50%. Future studies to correlate PD-L1 expression and treatment outcomes with checkpoint inhibitors are underway. Searching for practical tests to assess for immune response markers from peripheral blood is warranted.
